# Phytoestrogen Metabolism by Adult Human Gut Microbiota

**DOI:** 10.3390/molecules21081034

**Published:** 2016-08-09

**Authors:** Pilar Gaya, Margarita Medina, Abel Sánchez-Jiménez, José Mᵃ Landete

**Affiliations:** 1 Departamento de Tecnología de Alimentos, Instituto Nacional de Investigación y Tecnología Agraria y Alimentaria (INIA), Carretera de La Coruña Km 7.5, Madrid 28040, Spain; pgaya@inia.es (P.G.); mmedina@inia.es (M.M.); 2 Departamento de Matemática Aplicada (Biomatemática), Facultad de Ciencias Biológicas, Universidad Complutense de Madrid, C/José Antonio Novais 12, Madrid 28040, Spain; abelsanchez@bio.ucm.es

**Keywords:** phytoestrogens, microbiota, equol, urolithins, enterolignans

## Abstract

Phytoestrogens are plant-derived polyphenols with a structure similar to human estrogens. The three main groups of phytoestrogens, isoflavones, ellagitannins, and lignans, are transformed into equol, urolithins, and enterolignans, respectively, by bacteria. These metabolites have more estrogenic/antiestrogenic and antioxidant activities than their precursors, and they are more bioavailable. The aim of this study was to analyze the metabolism of isoflavones, lignans and ellagitannins by gut microbiota, and to study the possible correlation in the metabolism of these three groups of phytoestrogens. In vitro fermentation experiments were performed with feces samples from 14 healthy adult volunteers, and metabolite formation was measured by HPLC-PAD and HPLC-ESI/MS. Only the microbiota of one subject produced equol, while most of them showed production of *O*-desmethylangolensin (*O*-DMA). Significant inter-subject differences were observed in the metabolism of dihydrodaidzein and dihydrogenistein, while the glucoside isoflavones and their aglycones showed less variability, except for glycitin. Most subjects produced urolithins M-5 and E. Urolithin D was not detected, while uroltithin B was found in half of the individuals analyzed, and urolithins A and C were detected in two and four subjects, respectively. Enterolactone was found in all subjects, while enterodiol only appeared in five. Isoflavone metabolism could be correlated with the metabolism of lignans and ellagitannins. However, the metabolism of ellagitannins and lignans could not be correlated. This the first study where the metabolism of the three groups together of phytoestrogen, isoflavones, lignans, and ellagitannins by gut microbiota is analyzed.

## 1. Introduction

Phytoestrogens are polyphenols found in high concentration in soya, flaxseed and other seeds, fruits, vegetables, cereals, tea, chocolate, etc. [1,2,3]. Although phytoestrogens are nonsteroidal, they have similarities in chemical structure to mammalian estrogens, show estrogenic activities in biological assays, and induce estrogen-like effects in mammalian systems [4]. They comprise several classes of chemical compounds (stilbenes, coumestans, isoflavones, ellagitannins, and lignans), which can have both estrogenic and antiestrogenic effects [5].

It has been demonstrated that polyphenols are extensively metabolized once they are absorbed through the gut barrier or, for the non-absorbed fraction and the fraction re-excreted in the bile, by the colonic microbiota [6]. Isoflavones, ellagitannins, and lignans are metabolized by intestinal bacteria to produce equol, urolithins, and enterolignans, respectively [5]. Equol, urolithin, and, enterolignans are more bioavailable, and they have more estrogenic/antiestrogenic and antioxidant activity than their precursors. Moreover, equol, urolithins, and enterolignans have anti-inflammatory, antiproliferative, and apoptosis-inducing effects [7].

Intervention studies have shown that the ability of the intestinal microbiota of some individuals to convert isoflavones, ellagitannins, and lignans into equol, urolithin and, enterolignans, respectively, may result in a reduced risk of hormone-dependent diseases [5,7,8,9,10]. Hence, the transformation of isoflavones, ellagitannins, and lignans by intestinal microbiota is essential in the protection against menopausal symptoms and certain chronic diseases, such as cancer, cardiovascular disease, and osteoporosis [8,9,10,11,12,13]. Although epidemiological and experimental evidence indicates that the intake of phytoestrogens in foods may protect against certain chronic diseases, discrepancies between the in vivo and in vitro assays with phytoestrogens have been observed. These discrepancies could be explained by the low bioavailability of phytoestrogens [5].

Setchell et al. [14,15] proposed that “the clinical effectiveness of soy protein in cardiovascular, bone, and menopausal health may be a function of the ability to biotransform soy isoflavones to the more potent estrogenic isoflavone, equol”, noting that this ability was dependent on the presence of intestinal bacteria capable of carrying out that metabolism. Phytoestrogen producing phenotypes proved to be stable; no conversion from equol producer phenotype to non-producer or vice versa has been observed [16]. These observations are also consistent with several studies of intestinal bacterial profiles in adults, in which the presence of particular bacterial profiles, characterized by molecular techniques, appear relatively stable over time [17].

On the other hand, phenolic compounds are also antimicrobial and can interact with the gut microbiota. Phytoestrogens might modulate the diversity of the gut microbiota due to their antimicrobial activity [18]. The inhibition of pathogens, or an increase of beneficial populations, might contribute to improving the health of the individual. Since polyphenols are extensively metabolized by the colonic microbiota, the aim of this study was to analyze, for the first time, the metabolism of the three groups together of phytoestrogens, isoflavones, lignans, and ellagitannins by gut microbiota and to study the possible correlation in their metabolisms. Accordingly, in vitro fermentation experiments were performed with feces from 14 healthy adult volunteers and the conversion of phytoestrogens to their derivatives was studied.

## 2. Results and Discussion

The bioavailability of dietary phytoestrogens influences the bioactivity of these compounds. Isoflavones, ellagitannins, and lignans derived from food occur mainly as glucosides and their intestinal absorption requires deglycosylation [6]. β-Glucosidases from gut microbiota are involved in this hydrolysis [19,20,21]. Feces from all the subjects assayed in the present work showed glucosidase activity higher than 150 µM p-nitrophenyl/mg of protein.

Bioactive aglycones are extensively conjugated during and after absorption through the gut barrier [6]. Polyphenols are conjugated to form *O*-glucuronides and sulphate esters. Virtually no free aglycones are found in plasma [22], except for particular flavonoids such as phloretin [23]. This conjugation first occurs in the gut barrier and then reaches the liver, where it is further metabolized [24]. The formation of anionic derivatives by conjugation with glucuronide and sulphate groups facilitates their urinary and biliary excretion, and explains their rapid elimination. Microbiota can deconjugate the glucuronide and sulphate groups excreted in the bile [25]. Hence, glucuronidase activity higher than 100 µM p-nitrophenyl/mg of protein, was demonstrated in all the samples analyzed in the present work. Later on, in the colon, the glycosylated, sulfated, and glucuronidated forms of phytoestrogens are deconjugated by bacterial enzymes, consequently, the reuptake of phytoestrogens is enhanced [26], and then subjected to further metabolism by the intestinal microbiota as discussed below [27,28,29].

### 2.1. Metabolism of Isoflavones

**Figure 1:**
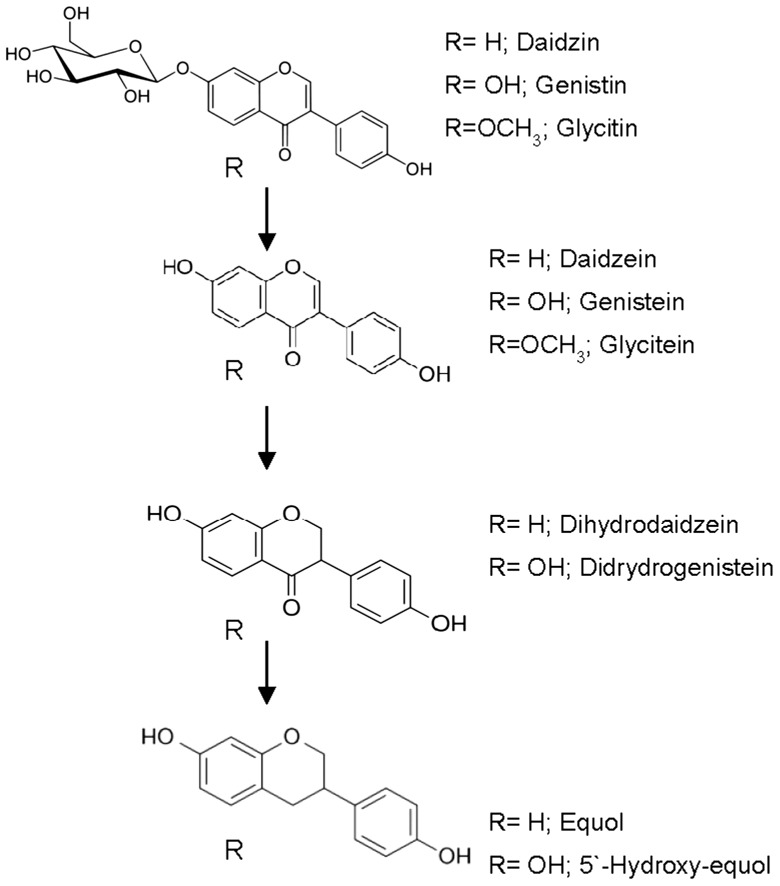
Metabolic pathway of isoflavones.

Soy isoflavones are found mainly as glycosides (daidzin, genistin, and glycitin) [30]. Their bioavailability requires the conversion to aglycones (daidzein, genistein, and glycitein) by means of β-glucosidase enzymes from bacteria [14,15] (Figure 1). A decrease in both the concentration of daidzin and daidzein was registered in the fermentation of feces from the 14 subjects investigated in the present work. Daidzin was greatly reduced by transformation to daidzein as a consequence of the β-glucosidase activity. Daidzein concentration also decreased in all individuals as a result of transformation into dihydrodaidzein (Table 1). The genistin virtually disappeared in all subjects. However, genistein remained, or even increased, as a consequence of the biotransformation of genistin into genistein.

Although bacteria with β-glucosidase activity are capable of the deglycosylation of daidzin, genistin, and glycitin, we previously observed that lactic acid bacteria and bifidobacteria have preference for the production of genistein from genistin, and daizein and/or glycitein production does not occur in many cases (Data not shown). One possible explanation is that β-glucosidase has more affinity for genistin, as already shown [31].

Main inter-individual differences in the metabolism of isoflavone glucoside were observed in the glycitin metabolism. As demonstrated in previous works [32], the glycitin concentration in fermented extracts was below those of daidzin and genistin. An increased content of glycitin was noted in subjects H5, H6, H7, H8, and H13 when compared with the control, this may be due to the conversion of malonylglycitin into glycitin through hydrolysis [33], although not as much as in subjects H2 and H10. On the contrary, the glycitin concentration decreased in subjects H1, H3, H4, H9, H11, and H14. A significant reduction in the concentration of glycitein was observed for all subjects with the exception of H5 and H10 (Table 1), who did not show a significant variation.

Some isoflavones, which are neither hydrolyzed nor absorbed in the intestine, reach the colon, together with isoflavones excreted into the intestine from enterohepatic circulation. There daidzein is metabolized via dihydrodaidzein into *O*-desmethylangolensin (*O*-DMA) or equol if the adequate microbiota is present. The dihydrodaidzein concentration increased greatly in subjects H2, H6, H8, H10, and H14, but the largest increase was observed in H6, who was the only equol producer (Table 1). This was a celiac, lactose intolerant woman. Feces from her sister, who is neither celiac nor lactose intolerant, did not contain equol. Imbalances in the intestinal microbiota have been associated with the celiac disease [34], differences in the intestinal microbiota of the sisters may be associated with the production of equol. The rest of the subjects did not show a significant increase in dihydrodaidzein production. With the exception of the woman who produced equol, no significant differences were observed in the metabolism of isoflavones between men and women. These results reflect the importance of dihydrodaidzein producing microorganisms for the production of equol.

Two distinct phenotypes of bacterial daidzein metabolism have been observed in human populations; equol producing individuals and non equol producers. Equol producing subjects represent only 7.2% in our study. This percentage is well below the 20%–30% of equol producing individuals in populations in Western countries, and 50%–60% in Asian populations [35]. We must bear in mind that the study is restricted to 14 individuals living in Madrid (Spain). These phenotypes appear to be stable in individuals over time [16], suggesting some degree of genetic predisposition. In contrast to the variability observed in different human populations, studies with small numbers of animals showed a high proportion of equol-producing animals such as rats and chimpanzees [36,37].

Colonization of germ-free rats with a fecal microbiota from a human subject with the capacity to convert daidzein to equol, resulted in the excretion of substantial amounts of equol in rat feces. On the other hand, equol was undetectable in the urine of rats colonized with a fecal microbiota from a low equol-producing subject [38]. Hence, the inability of some subjects to produce equol is a consequence of the lack of gut microbiota with the capacity to convert daidzein to equol.

Described equol-producing bacteria belong to the Coriobacteriaceae family, with the exception of Lactococcus 20–92 [39]. The fact that only a limited number of bacterial strains capable of degrading daidzein and producing equol have been isolated and identified may be due to the need for anaerobic culture conditions and specific medium constituents. Moreover, a large percentage of intestinal bacteria are unculturable

*O*-DMA concentration increased in all extracts examined with the exception of feces from subjects H1 and H12, which did not show *O*-DMA production. Unlike the equol producer phenotype, the *O*-DMA producer phenotype is much more common among the population. It is interesting to note that equol producer H6 showed a lower production of *O*-DMA, demonstrating that the metabolic pathway is diverted to the production of equol [40].

The ability of equol to play a role in the treatment of estrogen or androgen-mediated diseases or disorders, such as menopause and breast, colon, bone, and prostate cancer has been extensively proposed [5,41,42]. Equol is able to bind both estrogen receptors α and β better than daidzein, dihydrodaidzein and *O*-DMA. Moreover, equol is more bioavailable, and has more antioxidant activity. The higher antioxidant activity of equol may be a result of its nonplanar structure which confers a greater flexibility for conformational changes, enabling this compound to penetrate more easily into the interior of the membrane and protein or lipid structures to prevent oxidative damage in situ in contrast to some of the other isoflavones which are more rigid in structure [43].

Genistein is metabolized as dihydrogenistein by intestinal bacteria. An increase in dihydrogenistein was observed in all subjects with the exception of H6 and H12, and the extracts from subjects H11 and H13 exhibited the highest production. A peak with molecular weight (257.0819 g/mol) identical to 5-hydroxy-equol (was found in most samples, but it was not found in the control. This suggests that the production of this compound could be more common than equol, although this result must be confirmed with the standard compound. Interestingly, 5-hydroxy-equol was reported to show an antioxidant activity superior to that of genistein [44]. As shown for other isoflavonoids such as equol, 5-hydroxy-equol which is also expected to bind to estrogen receptors, preferably to estrogen receptor β [41]. Whereas 5-hydroxy-equol is produced from genistein via dihydrogenistein [45], the C-ring cleavage yielding 6′-hydroxy-*O*-desmethylangolensin and subsequently 2-(4-hydroxyphenyl) propionic acid is catalyzed by some intestinal bacteria such as Eubacterium ramulus [46]. To our knowledge, this is the first study where 5-hydroxy-equol has been detected as a product of human or intestinal microbiota.

### 2.2. Metabolism of Ellagitannins

Ellagitannins present in food are metabolized into ellagic acid by gut microbiota. This transformation is facilitated by physiological pH. Ellagitannins and ellagic acid are not usually absorbed, and they must be transformed to urolithins (dibenzopyran-6-one metabolites) before their absorption [47,48]. Gut microbiota transform the ellagic acid to urolithins by lactone-ring cleavage, decarboxylation and dehydroxylation reactions [49,50]. Ellagic acid is gradually metabolized in the intestine, starting with urolithin M-5 and finally urolithin A and urolithin B after successive dehydroxylation reactions by gut microbiota.

All the fermented samples with the only exception of the subject H1 and the control, presented at least two of the urolithins A–E and M-5. Worth noting is subject H5, who produced four urolithins (A, B, E, and M-5) (Table 2). Most of the intestinal microbiota of the adult subjects analyzed showed urolithin M-5 and E production. However, we did not find urolithin D production. Ellagic acid decreased in samples from all subjects after incubation, except in subject H1, although around 90% of ellagic acid was not consumed or transformed in the rest of subjects.

**Figure 2:**
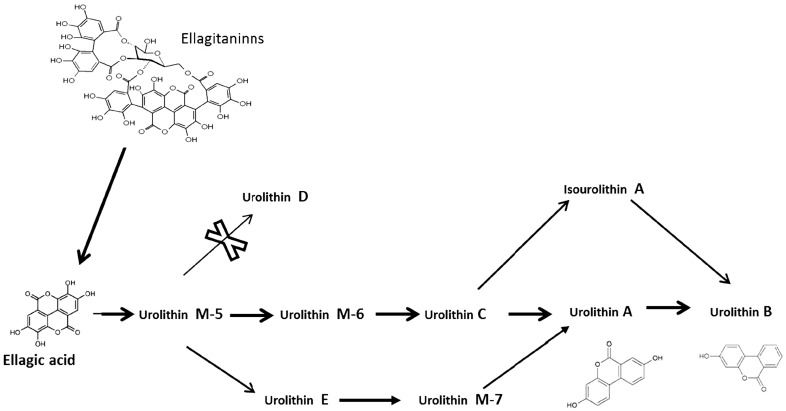
Metabolism of ellagitannins and ellagic acid by intestinal microbiota.

As mentioned above, urolithin M-5 is the first step in urolithin production from ellagic acid, followed by urolithin D, urolithin E and/or urolithin M6 (the last not analyzed in the present work) [51]. Our results, in concordance with others authors [51], suggest the transformation of urolithin M-5 into urolithins E and M-6, and further processing of urolithin M-6 into urolithin C by the intestinal microbiota (Figure 2). Both pathways occur in the intestine of human adults. While the processing of urolithin M-5 into urolithin D was not observed in the different samples analyzed, urolithin M-5 and urolithin E appeared in the analysis of most of the subjects, with the exception of H1 and H10 where none were detected, this could be explained by the transformation of urolithin M-5 into urolithin E. Only in subjects H4 and H14 did urolithin E appear, possibly, due to the total transformation of urolithin M-5.

No significant differences were observed in the metabolism of ellagitannins by the microbiota of men and women. However, considerable inter-individual differences were registered, identifying “high and low metabolite producers” in each group following the fermentation assays. The highest urolithin production was associated with urolithin C by subject H12 (16.633 mg/L). In subjects H2 and H10 a high production of urolithin B was observed. Urolithin A production was only registered from subjects H5 and H8. Urolithin B production by gut microbiota was observed in seven subjects (H2, H4–8 and H10), whereas four subjects showed urolithin C production (H4, H10, H12 and H13) (Table 2).

Urolithin A was detected together with urolithin E, whereas urolithin C did not appear. Hence, the metabolic pathway would be the route suggested by Garcia-Villalba et al. [51]; by which urolithin E is transformed into urolithin A via urolithin M7. In addition to the possible transformation of urolithin A into urolithin B in subjects H5 and H8, subjects H2, H4, H6, H7, and H10 showed production of uroltihin B, but urolithin A did not appear: transforming isourolithin A (not detected) into urolithin B has been suggested by Garcia-Villalba et al. [51]. More studies, and studies at different times, would be needed to confirm these metabolic routes. Still, we must consider the possible overlap of different bacterial groups capable of presenting different types of ellagitannins metabolism. For example, to explain the coincidence of urolithin C and urolithin E.

González-Barrio et al. [52] observed the catabolism of ellagitannins with the appearance of urolithin A-*O*-glucuronide and urolithin B-*O*-glucuronide in urine collected 7–48 h after raspberry consumption. We did not analyze the urolithin glucuronides, although, high glucuronidase activity was demonstrated in the microbiota of all subjects analyzed as mentioned above. Urolithin A was positively correlated to *Gordonibacter* in feces, whereas excretion of isourolithin A and/or urolithin B was inversely correlated to both [53]. The relationship between *Gordonibacter* and urolithin A found in vivo was also confirmed in vitro by these authors. A search for the bacteria involved in urolithin production would be of great interest.

### 2.3. Metabolism of Lignans

**Figure 3:**
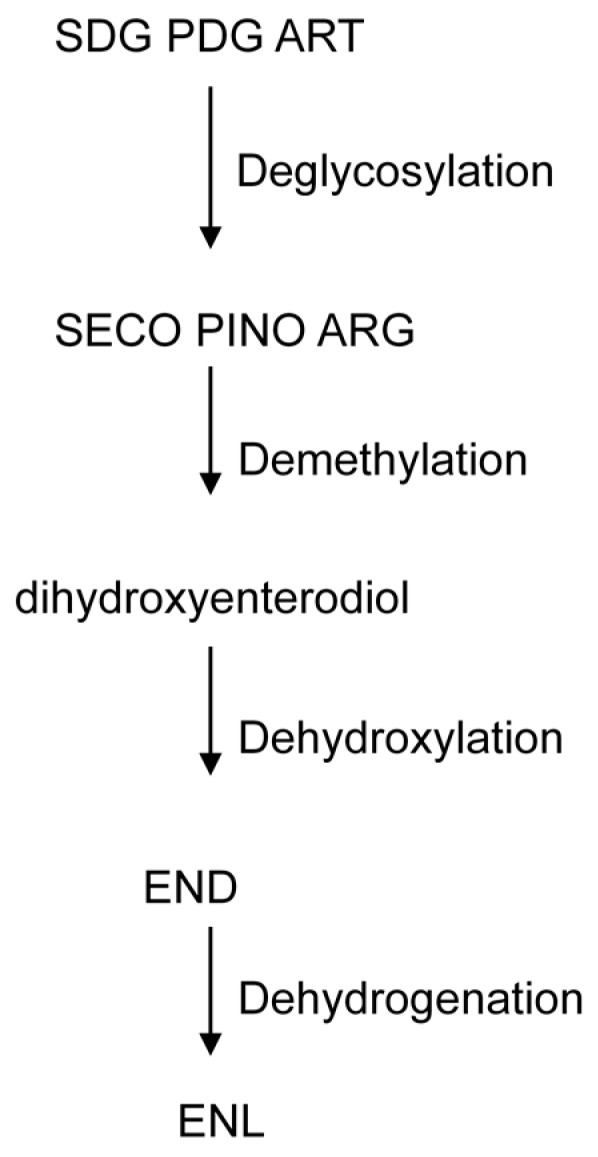
Metabolism of the lignans by intestinal microbiota. Pinoresinol diglucoside (PDG), arctiin (ART) secoisolariciresinol diglucoside (SDG), secoisolariciresinol (SECO), pinoresinol (PINO), arctigenin (ARG), enterodiol (END) and enterolactone (ENL).

The bioactivity of the dietary lignans depends on their transformation by gut bacteria in the colon [54]. Several authors have described the transformation by bacterial community of plant lignans into enterodiol and enterolactone [55,56]. Microbiota hydrolyze the sugar moiety of pinoresinol diglycoside (PDG), secoisolariciresinol-diglycoside (SDG) and arctiin to release pinoresinol, secoisolariciresinol (SECO) and artigenin [57,58] (Figure 3). SECO concentration was increased at least 10-fold in the majority of samples assayed in the present work, in concordance with an important decrease in SDG concentration (Table 3).

Low concentrations of pinoresinol and matairesinol were detected in the control, and artigenin was not detected. Matairesinol increased significantly with the presence of microbiota in all the samples analyzed. Artigenin and pinoresinol concentrations increased in the majority of samples, with the exception of feces from subject H1 where neither pinoresinol nor artigenin were detected, and from subject H9 who showed no signs of artigenin. In lignan extract, lignans are found mainly in the form of glycosides. For this reason, we did not observe matairesinol, pinoresinol, and artigenin in the control sample. However, the concentration of these compounds increased significantly in the presence of microbiota via deglycosylation of arctiin, PDG and SDG.

Deglycosylation is followed by demethylation. Demethylation of SECO by microbiota produced dihydroxyenterodiol (2,3-bis(3,4-dihydroxybenzyl)-1,4-butanediol). An increase in the dihydroxyenterodiol concentration was found in samples from all subjects, confirming the production of enterolignans by the intestinal microbiota. *Butyribacterium methylotrophicum*, *Eubacterium callanderi*, *Eubacterium limosum*, *Ruminococcus productus* and *Peptostreptococcus productus* were identified by Clavel et al. [59] in dihydroxyenterodiol production. Lariciresinol has been described to increase significantly in all subjects analyzed, formed from syringaresinol and pinoresinol, following the production of pinoresinol from PDG [60].

Demethylation is followed by dehydroxylation to produce enterodiol. Production of enterodiol was found in extracts from 5 out of the 14 adult subjects investigated. Two reactions of demethylation and dehydroxylation are needed to transform matairesinol into enterolactone, with dihydroxyenterolactone (2,3-bis(3,4 dihydroxybenzyl) butyrolactone) being found after demethylation reactions [58]. Borriello et al. [61] showed how matairesinol is metabolized to enterolactone by human intestinal microbiota, while Clavel et al. [59] demonstrated how *Ruminococcus productus* catalyzes the demethylation of matairesinol to dihydroxyenterolactone. However, we did not detect any peak with molecular weight similar to dihydroxyenterolactone by HPLC-ESI/MS analyses. This suggests that this metabolic pathway does not appear, contrary to what we observed with the metabolism of dihydroxyenterodiol mentioned above.

Dehydroxylation is followed by dehydrogenation from enterodiol to produce enterolactone. Production of enterolactone was registered in all extracts, whereas production of enterodiol was found in extracts from 5 out of the 14 adult subjects investigated (Table 3), suggesting the rapid transformation of enterodiol to enterolactone. It is noteworthy that all subjects produced enterolactone. No gender-related differences were observed in the metabolism of lignans.

Although some interindividual differences could be observed in enterolignan production, the gut microbiota from adult humans presented a similar behavior in the metabolism of lignans. Factors controlling the bioactivation of lignans in the large intestine are diet, transit time, intestinal redox state and, most importantly, the composition and activity of the colonic microbiota [61,62]. Due to variations in these factors, differences among individuals were observed in lignan bioactivation in urine, fecal, and blood samples, leading to a subdivision of enterolignan producers into weak, moderate, and strong phenotypes [62,63].

### 2.4. Correlation between Pairs of Groups of Metabolites

We excluded equol, urolithin A, urolithin C, and enterodiol due to the low number of observations among samples. CCA between isoflavones and ellagic acid yielded one significant function (Wilks’s λ = 0.00022, *F*(27, 6.48) = 4.08, *p* = 0.036) with squared canonical correlation (rc^2^ = 0.99). Based on structural coefficients it seemed to exist associations among daidzin (rs = −0.47), genistin (rs = −0.57), glycitin (rs = −0.55), urolithin B (rs = −0.45), and urolithin M-5 (rs = −0.53), which were all of them positively related, and a negative relationship between these metabolites and urolithin E (rs = 0.66). CCA between isoflavones and lignans yielded one significant function (Wilks’s λ = 0.01589, *F*(15, 16.96) = 2.45, *p* = 0.039) with rc^2^ = 0.84. Based on structural coefficients it seemed that there are associations among genistin (rs = −0.61), genistein (rs = −0.58), glycitein (rs = −0.61), glycitin (rs = −0.73), and arctigenin (rs = −0.66), which were all of them positively related, and a negative relationship between these metabolites and SECO (rs = 0.65) and matairesinol (rs = 0.49). Finally, there was no significant CCA function between ellagic acids and lignans.

Factor analysis of the entire set of metabolites yielded six components based on eigenvalues criteria λ > 1 (Table 4). Only from the first three factors, that accumulated more than 60% of the explained variance (Table 4), we found groups of metabolites with strong associations among them (Table 5). This way, rotate loadings of the first factor showed a great correlation among daidzin, genistin, glycitin, urolithin B, and urolithin M-5, in the same direction, and with SECO in the opposite direction (Table 5). Second factor revealed a positive relationship between daidzein, genistein, and glycitein, which also correlated with matairesinol in the opposite direction. Enterolactone and arctigenin showed a strong association within third factor. The three remainder factors, that accumulated only 26% of the explained variance, were less useful in order to find metabolites relationships as they comprised variables with high rotate loadings (for example DHD and pinoresinol in the fourth factor) and variables with moderate loadings (see urolithin E in factors 4, 5, and 6). Conclusion from these last three factors would not be too accurate.

## 3. Experimental Section

### 3.1. Chemicals and Solvents

Standardized extracts of soybean and flax were used. SoyLifes EXTRA, an isoflavone extract from soybean germs, and LinumLifeTM EXTRA, a lignan extract from flax, were provided by Frutarom Netherlands BV (Veenendaal, The Netherlands). The solvents used, methanol, acetic acid and acetonitrile were of HPLC grade (LabScan, Gliwice, Poland). The standard compounds, secoisolariciresinol (SECO), enterodiol, enterolactone, ellagic acid, matairesinol, artigenin, and pinoresinol were HPLC grade and purchased from Sigma-Aldrich (St. Louis, MO, USA). Daidzein, daidzin, equol, genistein, and genistin were purchased from LC Laboratories (New Boston Street, Woburn, MA, USA). Urolithin A, urolithin B, urolithin C, urolithin D, and urolithin E were purchased from Dalton pharma (Wildcat, Rd., Toronto, ON, Canada). Dihydrodaidzein and dihydrogenistein were purchased from Toronto Research Chemicals (Toronto, ON, Canada). Stock solutions of phytoestrogens were prepared in DMSO (Sigma-Aldrich) in a concentration of 10 mg/L.

### 3.2. Collection of Human Fecal Samples

Fecal samples were donated by 14 healthy volunteers (nine women and five men) aged 26–67, who consumed a non-specified western diet. They had no history of gastrointestinal disease or any chronic disease with the exception of H6. They were all non-smokers, and had not used antibiotics in the three months before sample collection. Female volunteers were neither pregnant nor lactating. The subject H6 is a celiac and lactose intolerant female. The volunteers were fully informed of the aims of the study and gave their written consent. All procedures involving human participants were in accordance with the ethical standards of the institutional and/or national research committee, as well as the 1964 Helsinki declaration and its later amendments or comparable ethical standards.

### 3.3. Phytoestrogen Fermentation Assay

Soybean extracts (4 g/L), flax extracts (2 g/L), and ellagic acid (100 mg/L) dissolved in DMSO were added to the growth medium (sterilized at 121 °C for 20 min) consisting of Wilkins-Chalgren anaerobe broth (Oxoid Ltd., Basingstoke, Hampshire, UK) containing 0.5 g/L of l-cysteine. The pH was adjusted to pH 7.4 with NaOH. Samples were processed within 30 min of defecation. Fecal suspensions were prepared by mixing freshly collected human fecal samples (1 g) in PBS (100 mL), follow by centrifugation at 200 g for 1 min. at 4 °C., then 100 µL of supernatant was added to 10 mL of Wilkins-Chalgren anaerobe broth with polyphenol extracts. The amount of DMSO did not affect bacteria growth. The fecal suspensions with the polyphenols were incubated in sealed jars (Oxoid Ltd.) under anaerobic conditions using AnaeroGen sachets (Oxoid Ltd.) at 37 °C for five days.

Wilkins-Chalgren anaerobe broth with polyphenols (Soybean extracts, flax extracts and ellagic acid), and without fecal suspension was used as control. The control was also incubated in sealed jars under anaerobic conditions at 37 °C for five days. All experiments were conducted in triplicate.

### 3.4. Extraction of Phytoestrogens

Bacterial suspensions were removed by centrifuge at 5000 *g* for 5 min. Phytoestrogens from 10 mL of Wilkins-Chalgren anaerobe broth with polyphenols and fecal suspension, as well as from the control with polyphenols and without fecal suspension, were extracted twice with 2 mL of diethyl ether and twice with 2 mL of ethyl acetate. The solvents were evaporated at room temperature under a N_2_ stream and the residue was dissolved in 300 µL methanol/water (50:50, *v*/*v*) and filtered through a 0.22 μm cellulose acetate filter (Millipore, Madrid, Spain), then transferred into HPLC vials and stored at −20 °C until analysis.

### 3.5. HPLC-PAD and HPLC-ESI/MS Analysis

Extracted samples were subjected to HPLC-PAD and HPLC-ESI/MS using a HPLC-PAD Beckman System Gold (Beckman Coulter Inc., Fullerton, CA, USA), comprising an autosampler module 508, a binary pump module 126, a diode array detector module 168, and 32 Karat Software chromatography manager. Separation of phenolic compounds was achieved on a reverse phase Nova-Pak C18 column (300 mm × 3.9 mm, 4 μm) (Waters, Barcelona, Spain). The analytical conditions were based on those described by Gaya et al. [64].

Mass spectra were obtained using a LC-MS Agilent 1200 (Palo Alto, CA, USA) chromatography system equipped with a quaternary pump (G1311A), a degasser (G1322A), an thermostated autosampler (G1367B), a thermostated column compartment (G1316A), a photodiode array detector (G1315B), and a quadrupole mass spectrometer (QTOF Agilent G6530A) with an electrospray ionization (ESI) interface and Masshunter Data Adquisition and Qualitative Analysis (B.40.0) as control software. Other ESI/MS parameters were as follows: range acquisition 100–1000 m/z, gas temperature 350 °C, gas flow 10 L/min, nebulizer 45 psig, sheath gas temperature 350 °C, sheath gas flow 11 L/min, capillary voltage 3500 V, and fragmentation voltage 120 V. The mass spectrometer operated in the negative ion mode.

Separation was carried out with the same column detailed above for the HPLC-PAD analysis at 30 °C. A volume of 25 μL was injected and elution was performed with a gradient consisting of solvent A (water/acetic acid, 98:2 *v*/*v*), solvent B (water/acetonitrile/acetic acid, 78:20:2 *v*/*v*/*v*) and solvent C (acetonitrile) at a flow rate of 1 mL/min. The gradient profile was 0 min (50% A, 50% B); 10 min (45% A, 45% B; 10% C); 20 min (40% A, 40% B; 20% C); 40 min (40% A, 40% B; 20% C); 45 (45% A, 45% B; 10% C); and 50 min (50% A, 50% B).

### 3.6. Identification and Quantification of Phenolic Compounds

Chromatographic peaks were identified by HPLC-ESI/MS and confirmed by comparison of retention times and characteristics of UV spectra with those of standards. Compounds for which standards were not available were tentatively identified according to their retention times, UV spectra by HPLC-PAD, and data of HPLC-ESI/MS analysis.

Quantification was made using the external standard calibration curves, with commercial standards. Five concentrations (1.0, 0.5, 0.25, 0.125, and 0.0625 mg/L) and three injections per level of each standard were used. *O*-desmethylangolensin (*O*-DMA) and urolithin M-5 could be quantified by the calibration curves of the most similar compounds dihydrodaidzein and urolithin D, respectively. Dihydroxyenterolactone, lariciresinol and secoisolariciresinol-diglycoside (SDG) were not quantified, though their areas were measured by HPLC-ESI/MS.

### 3.7. Determination of β-Glucosidase and β-Glucuronidase Activities

Fecal suspensions were grown in 10 mL of Wilkins-Chalgren anaerobe broth under anaerobic conditions for 24 h at 37 °C. The medium was centrifuged at 4000× *g* for 3 min and the pellets containing the cells were re-suspended in a phosphate buffer (0.2 M, pH 6.8). Suspensions were incubated separately with p-nitrophenyl-β-d-glucopyranoside (2.5 mM) and p-nitrophenyl-β-d-glucuronide (2.5 mM) (Sigma-Aldrich, St. Louis, MO, USA) in aerobic conditions for 30 min at 37 °C. Release of p-nitrophenol was measured using a spectrophotometer (Beckman DU 650, Fullerton, CA, USA) at 405 nm, before and after incubation [65].

### 3.8. Correlation Analysis

We performed canonical correlation analysis (CCA) between pairs of groups of metabolites (isoflavones, ellagic acids, and lignans) to evaluate the multivariate shared relationship between them. Metabolites with structural coefficients (rs) with absolute value above 0.45 were considered to correlate with each other. To search for associations among all metabolites we implemented a factor analysis with Varimax rotation. All analysis were performed using SPSS v22 software. Significance level (α) was set to 0.05 in all cases.

## 4. Conclusions

To our knowledge, this is the first study where the phytoestrogen metabolism of the three groups together of, isoflavones, lignans, and ellagitannins by gut microbiota is analyzed and inter-individual differences in the microbial conversion of phytoestrogens reported. Our work, in concordance with others woks, demonstrate that both isoflavones and lignans are found mainly in the form of glycosides in food, and after microbial deglycosylation the phytoestrogens are subjected to further metabolism by intestinal microbiota. Only the microbiota present in feces from one individual produced equol, while most showed production of *O*-DMA. Almost all individuals produced some kind of urolithins, all produced enterolactone and showed a similar behavior in the metabolism of other lignans. It is necessary to identify the key gut microorganisms responsible for the conversion of plant constituents to biologically active derivatives, and the factors that determine their occurrence in the gastrointestinal tract.

## Figures and Tables

**Table 1:** Metabolism of isoflavones (µM) by adult human gut microbiota.

Comp.	Control	H1	H2	H3	H4	H5	H6	H7	H8	H9	H10	H11	H12	H13	H14
**Equol**	nd	nd	nd	nd	nd	nd	1.0	nd	nd	nd	nd	nd	nd	nd	nd
**Daidzein**	3158.2	1040.5	993.1	2348.1	2505.4	3076.4	1739.5	1873.5	1200.1	1081.6	2584.4	1564.1	1408.0	306.6	710.1
**Daidzin**	4437.9	39.2	445.4	95.3	31.3	39.7	50.3	127.6	68.1	42.3	43.6	190.0	355.8	244.2	220.4
**Genistin**	3032.2	nd	210.1	nd	nd	nd	20.6	nd	36.8	nd	45.8	110.8	81.4	44.6	33.3
**Genistein**	1877.7	2009.6	1522.4	4103.0	2249.0	4158.0	3241.0	1713.4	1615.7	1816.4	4144.6	2024.1	2561.2	917.3	1176.7
**Glycitein**	829.5	80.9	327.1	547.9	498.8	846.1	526.7	119.7	197.4	130.7	824.2	244.8	268.4	39.2	175.5
**Glycitin**	101.9	49.8	509.5	75.7	95.4	112.5	158.3	185.1	200.8	67.8	286.4	90.5	234.7	161.5	83.3
**DHD**	154.8	163.4	455.5	172.1	167.4	201.5	1812.8	134.0	273.6	190.9	295.2	136.8	146.6	152.1	358.3
**DHG**	23.6	25.4	71.2	49.0	63.8	52.7	21.7	29.1	128.0	35.7	103.2	408.0	21.3	315.7	78.2
***O*-DMA**	nd	nd	1264.1	89.8	1373.1	1520.9	66.1	1164.6	109.6	108.9	135.3	854.9	nd	74.6	88.6

nd: not detected; (DHD, dihydrodaidzein; DHG, dihydrogenistein; *O*-DMA, *O*-desmethylangolensin).

**Table 2:** Metabolism of ellagic acid (µM) by adult human gut microbiota.

Comp.	Control	H1	H2	H3	H4	H5	H6	H7	H8	H9	H10	H11	H12	H13	H14
**Urolithin A**	0	nd	nd	nd	nd	0.105	nd	nd	1.739	nd	nd	nd	nd	nd	nd
**Urolithin B**	0	nd	33.472	nd	0.108	3.079	0.251	0.080	1.720	nd	20.180	nd	nd	nd	nd
**Urolithin C**	0	nd	nd	nd	14.072	nd	nd	nd	nd	nd	24.231	nd	68.440	14.792	nd
**Urolithin D**	0	nd	nd	nd	nd	nd	nd	nd	nd	nd	nd	nd	nd	nd	nd
**Urolithin E**	0	nd	0.509	1.633	2.030	4.250	3.339	0.088	0.706	0.335	nd	1.277	0.2586	4.293	0.208
**Urolithin M-5**	0	nd	15.064	0.214	nd	6.628	10.068	4.192	0.283	2.785	nd	3.243	5.69	3.988	nd

nd: not detected.

**Table 3:** Metabolism of lignans (µM) by adult human gut microbiota.

Comp.	Control	H1	H2	H3	H4	H5	H6	H7	H8	H9	H10	H11	H12	H13	H14
**Enterodiol**	nd	nd	4.84	nd	14.74	nd	5.77	nd	0.56	nd	14.77	nd	nd	nd	nd
**DHend**	nd	↑↑	↑	↑	↑↑	↑	↑	↑	↑↑	↑↑	↑↑↑	=	↑↑	↑	↑
**Enterolactone**	nd	7.06	29.01	8.61	26.89	24.03	11.88	26.55	67.04	18.14	34.90	15.95	9.25	16.89	60.95
**SECO**	70.88	685.83	334.74	733.73	776.09	684.45	842.82	792.43	900.69	846.70	764.19	474.85	647.90	848.64	379.05
**Matairesinol**	0.92	23.15	25.56	14.72	29.06	26.68	17.72	35.44	30.07	26.09	15.90	14.47	23.12	37.29	37.15
**Pinoresinol**	0.02	nd	10.33	4.76	9.74	113.79	5.29	14.00	12.65	43.70	7.42	41.72	40.09	2.26	8.14
**Arctigenin**	nd	nd	5.93	0.81	4.31	2.94	1.84	1.28	5.37	nd	2.74	0.61	0.616	0.89	0.084
**Lariciresinol**	nd	↑↑	↑↑	↑↑	↑↑	↑↑	↑↑	↑↑	↑↑	↑↑	↑↑	↑↑	↑↑	↑↑	↑↑
**SDG**	nq	↓↓↓	↓↓↓	↓↓↓	↓↓↓	↓↓↓	↓↓↓	↓↓↓	↓↓↓	↓↓↓	↓↓↓	↓↓↓	↓↓↓	↓↓↓	↓↓↓

nd: not detected; nq: Not quantified; DHend, dihydroxyenterodiol; SECO, secoisolariciresinol; SDG, secoisolariciresinol-diglycoside; increases the concentration (area) low ↑, medium ↑↑ or high ↑↑↑, decreases the concentration (area) low ↓, medium ↓↓ or high ↓↓↓.

**Table 4:** Eigenvalues, percent of explained variance of each factor and cumulative percentage of variance explained.

Factor	Eigenvalue	% Variance	Cumulative
1	4.66	27.43	27.43
2	3.93	23.14	50.56
3	1.91	11.21	61.78
4	1.81	10.62	72.39
5	1.56	9.16	81.56
6	1.07	6.28	87.84
7	0.72	4.25	92.08
8	0.53	3.11	95.19
9	0.40	2.35	97.54
10	0.22	1.28	98.82
11	0.16	0.96	99.78
12	0.03	0.17	99.95
13	8.25 × 10^3^	0.05	100.00
14	4.86 × 10^−11^	0.00	100.00
15	3.17 × 10^−11^	0.00	100.00
16	1.18 × 10^−12^	0.00	100.00
17	0.00	0.00	100.00

**Table 5:** Factor loadings and communalities for each variable under Varimax rotation.

Metabolites	Factor						Communalities
	1	2	3	4	5	6	
**Daidzein**	−0.23	**0.82**	0.13	0.43	−0.04	0.16	0.95
**Daidzin**	**0.83**	−0.39	−0.12	0.03	−0.02	−0.08	0.87
**Genistin**	**0.95**	−0.07	0.09	−0.04	0.02	−0.24	0.98
**Genistein**	−0.16	**0.93**	−0.12	0.13	0.10	0.15	0.95
**Glycitein**	−0.01	**0.88**	0.20	0.28	0.16	0.07	0.92
**Glycitin**	**0.83**	0.05	0.41	−0.06	0.18	0.13	0.91
**DHD**	**0.04**	0.16	−0.02	−0.28	**0.82**	0.17	0.80
**DHG**	**0.10**	−0.15	−0.03	0.00	−0.08	**−0.97**	0.97
***O*-DMA**	**0.24**	0.11	0.28	**0.81**	−0.02	0.01	0.80
**Urolithin B**	**0.80**	0.26	0.40	−0.06	0.02	0.10	0.88
**Urolithin E**	−0.27	0.07	−0.05	0.45	0.67	−0.41	0.91
**Urolithin M-5**	**0.70**	−0.05	−0.08	0.28	0.61	0.14	0.98
**Enterolactone**	−0.05	−0.25	**0.79**	−0.08	−0.25	0.01	0.76
**SECO**	**−0.73**	0.09	0.13	−0.16	0.33	0.04	0.69
**Matairesinol**	−0.18	−0.82	0.35	0.27	−0.03	0.14	0.92
**Pinoresinol**	−0.05	0.23	−0.18	**0.81**	−0.05	−0.02	0.74
**Arctigenin**	0.34	0.18	**0.82**	0.18	0.21	0.02	0.89
